# Comparative analysis of rhizosphere soil physiochemical characteristics and microbial communities between rusty and healthy ginseng root

**DOI:** 10.1038/s41598-020-71024-8

**Published:** 2020-09-25

**Authors:** Xingbo Bian, Shengyuan Xiao, Yan Zhao, Yonghua Xu, He Yang, Lianxue Zhang

**Affiliations:** 1 College of Chinese Medicinal Materials, Jilin Agricultural University, Changchun, 130118 Jilin Province China; 2 National and Local Joint Engineering Research Center for Ginseng Breeding and Development, Changchun, 130118 China

**Keywords:** Ecology, Microbiology, Plant sciences

## Abstract

Ginseng rusty root (GRR) symptom is one of the primary diseases of ginseng. There has been a problem of ginseng rusty root, leading to a severe decline in the quality of ginseng. To clarify the relationship between root symptoms of ginseng rust and soil, the physical and chemical properties, enzyme activity, community structure and microbial diversity of GRR and healthy ginseng (HG) rhizosphere soil were analyzed and compared. The pH and redox potential (Eh) of GRR soil decreased, and the contents of total phosphorus (TP), available phosphorus (AP), and available potassium (AK) decreased. The activity of catalase and phosphatase and invertase was lower than that of HG groups. Besides, the microbial community of GRR rhizosphere soil changes much, and its abundance and diversity are significantly reduced. The community structure of GRR rhizosphere soil also shows apparent differences, and the samples of the HG group gathered together, and the samples of the GRR group were dispersed. In general, GRR was closely associated with decreases in soil pH and Eh; decreases in TP, AP, and AK; decreases in the activity of several enzymes. Additionally, it is strongly associated with an increase in pathogenic microorganisms such as *Ilyonectria* and a reduction of beneficial microorganisms such as Tremellomycetes Acidobacteria subgroup 6 and Gemmatimonadetes.

## Introduction

Ginseng (*Panax ginseng* Mayer) is an essential medicinal material in China. Because of the relatively high planting benefits of ginseng and the limitation of planting soil, ginseng production areas are relatively concentrated. Moreover, in recent years, the scale of cultivated ginseng in the cutting forests has been shrinking; it is increasingly essential to produce high-quality ginseng. One of the significant factors affecting the ginseng quality is the various diseases that occur during the growth of ginseng.

Rusty root symptom is one of the central diseases in cultivation and production. It produces reddish-brown spots on the periderm of ginseng roots. With the increase of cultivation years, the patch may gradually expand, which will lead to the decline of commodity-grade and ginseng quality. At present, the mechanism of rust root is not precise. Whether ginseng rusty root is an infectious disease or a non-infectious physiological disease is still controversial, so some scholars have distinguished it^1^. Previous studies have shown that American ginseng (*Panax quinquefolius* L.) rusty root is the defense mechanism of ginseng itself, which is due to the invasion of some fungi that stimulates the production of phenolic compounds in ginseng^2,3^. Besides, some research results suggest that the induction of rusty root by fungal complex is the cause of rust roots in ginseng^4^. A variety of enzymes were used to hydrolyze plant structural materials and make ginseng roots rusty. Fe^3+^, combined with pectinase, had a synergistic effect on the formation of rust roots in ginseng^5^. Current studies suggest that ginseng rusty root symptom is related to soil acidity and alkalinity, water content, and metal element content, as well as the accumulation and oxidation of phenolic compounds in roots.

Soil microorganism is the most active part of the soil, driving force of soil material transformation and nutrient cycling. It participates in various soil processes, such as decomposition of soil organic matter, the formation of humus, transformation, and circulation of soil nutrients. Therefore, the composition and diversity of soil communities are closely related to soil function and plant health. Microorganisms in the soil are often the leading cause of plant diseases^6,7^. These previous studies focused on the soil enzyme activity and nutrient characteristics of different tree species and their relationship with the incidence index of GRR. Besides, some scholars explored the soil characteristics of several ginseng farms with GRR to explore the relationship between GRR and soil characteristics^8,9^. No data, however, are available on healthy ginseng and ginseng of rusty root on the same land on microbial community structure and enzyme activity in the plant rhizosphere.

In previous studies, the roots of GRR were collected, and fungi were isolated from the lesions to reveal the pathogen causing GRR^4^. Based on this phenomenon, it is hypothesized that some microbial communities should be closely related to GRR. By comparing to the rhizosphere soil of HG, any changes in the microbial communities of GRR may be explored.

In this study, we investigated the differences in nutrient content, enzyme activity, and other physical and chemical properties between GRR and HG soils. pH, redox potential, catalase, phosphatase, urease, invertase activities, and soil phenolic acids were measured. In addition, the 16S and ITS rRNA gene fragment obtained directly from soil samples were sequenced to study the microbial community structure and diversity in soil samples.

## Results

### Chemical properties of soil

Count the degree of disease of 100 ginsengs and calculate the rusty root index based on the formula (Table S1). Calculated by formula, the rusty root index is 0.7725, and this is a ginseng farm with very severe ginseng rusty root (Fig. S1).

**Figure 1:**
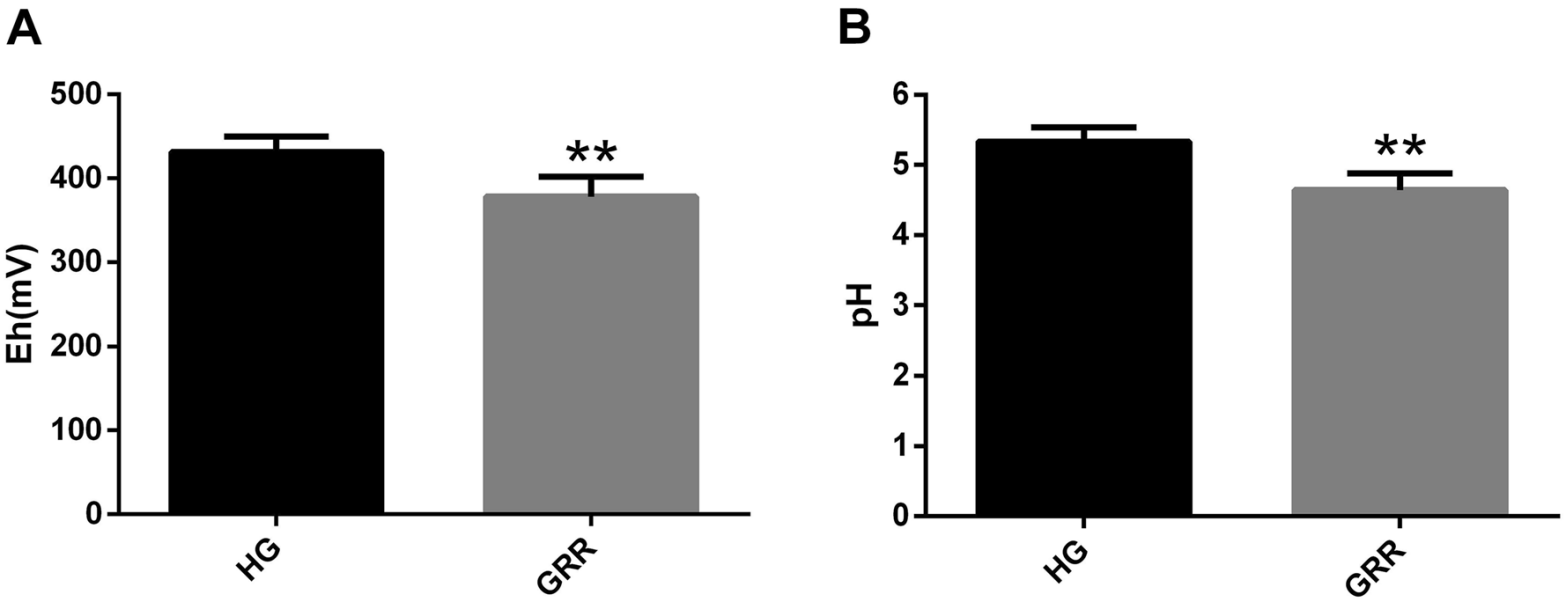
Chemical properties of soil. (**A**) Soil redox potential; (**B**) pH. HG: healthy ginseng soil groups; GRR: ginseng rusty root symptom soil groups. Letters indicate significant differences among different soil samples by ANOVA at *P* ** < 0.01 versus HG group (n = 6 in each group).

In soil pH measurement, we found that the ginseng planting of soil with dangerous rusty root phenomenon decreased significantly (Fig. 1A). In soil Eh measurements, GRR soils decreased compared considerably to soils of healthy ginseng (Fig. 1B).

**Figure 2:**
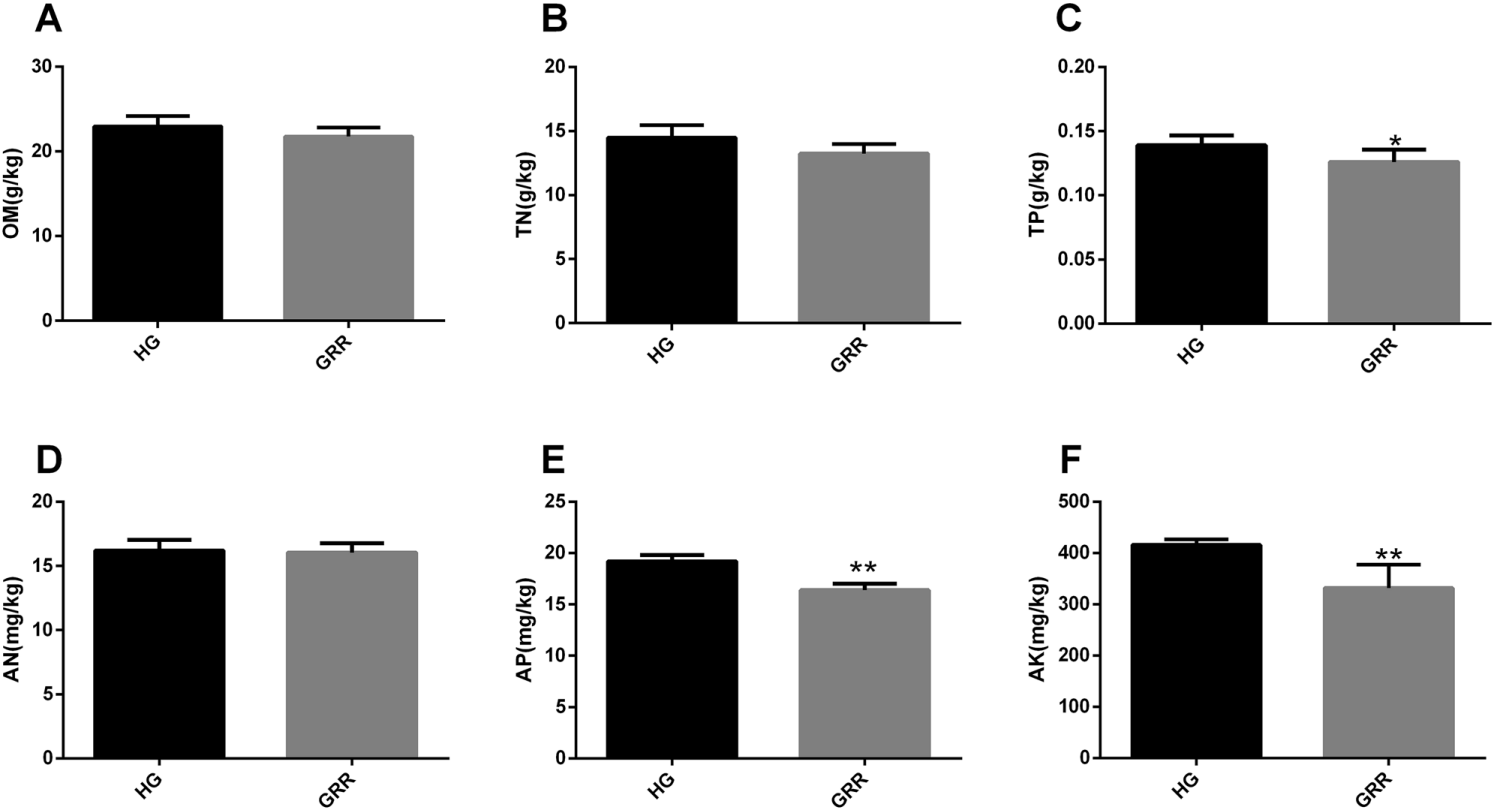
Nutrient content of soil. (**A**) Soil organic matter; (**B**) total nitrogen; (**C**) total phosphorus; (**D**) available nitrogen; (**E**) available phosphorus; (**F**) available potassium. HG: healthy ginseng soil groups; GRR: ginseng rusty root symptom soil groups. Letters indicate significant differences among different soil samples by ANOVA at *P* * < 0.05, *P* ** < 0.01 versus HG group (n = 6 in each group).

The nutrient content of the soil was also analyzed. The results showed that there was no significant difference in organic matter (OM), total nitrogen (TN), and available nitrogen (AN) between GRR soil and HG soil (Fig. 2A–C). Interestingly, however, tests of total phosphorus (TP), available phosphorus (AP), and available potassium (AK) showed a significant reduction in GRR soil compared to HG soil (Fig. 2D–F).

### Enzyme activity of soil

**Figure 3:**
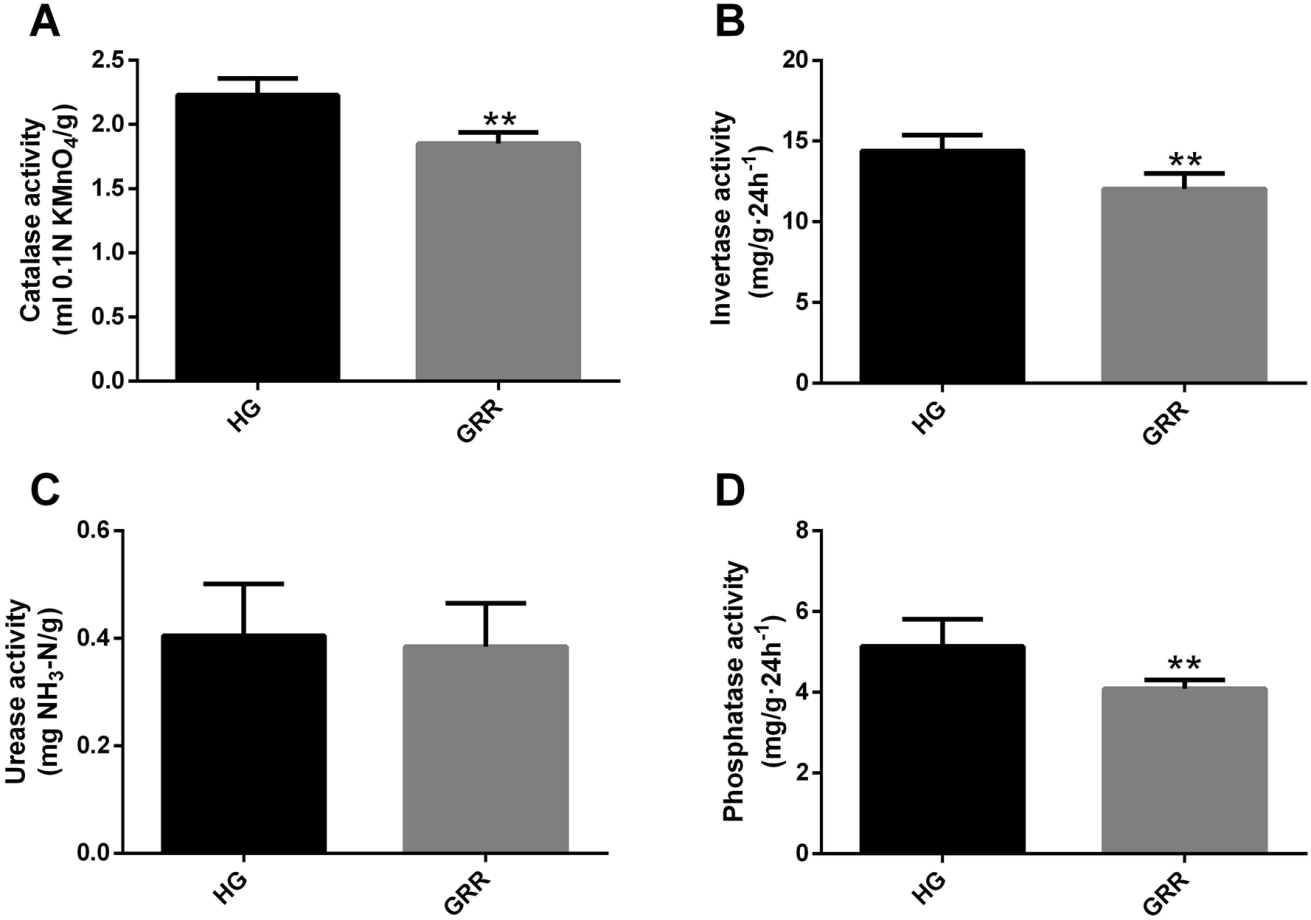
Soil enzyme activity. (**A**) Catalase activity; (**B**) invertase activity; (**C**) urease activity; (**D**) phosphatase activity. HG: healthy ginseng soil group; GRR: ginseng rusty root symptom-high soil group. Letters indicate significant differences among different soil samples by ANOVA at *P* ** < 0.01 versus HG group (n = 6 in each group).

Catalase, invertase, urease, and phosphatase of the two groups were compared. Among them, the activity of invertase, catalase and phosphatase in group GRR were significantly lower than those in the soil of healthy ginseng (Fig. 3A, B, D). However, there was no significant difference in urease activity among the two groups (Fig. 3C).

### Microbial diversity and richness in GRR and HG rhizosphere soils

**Figure 4:**
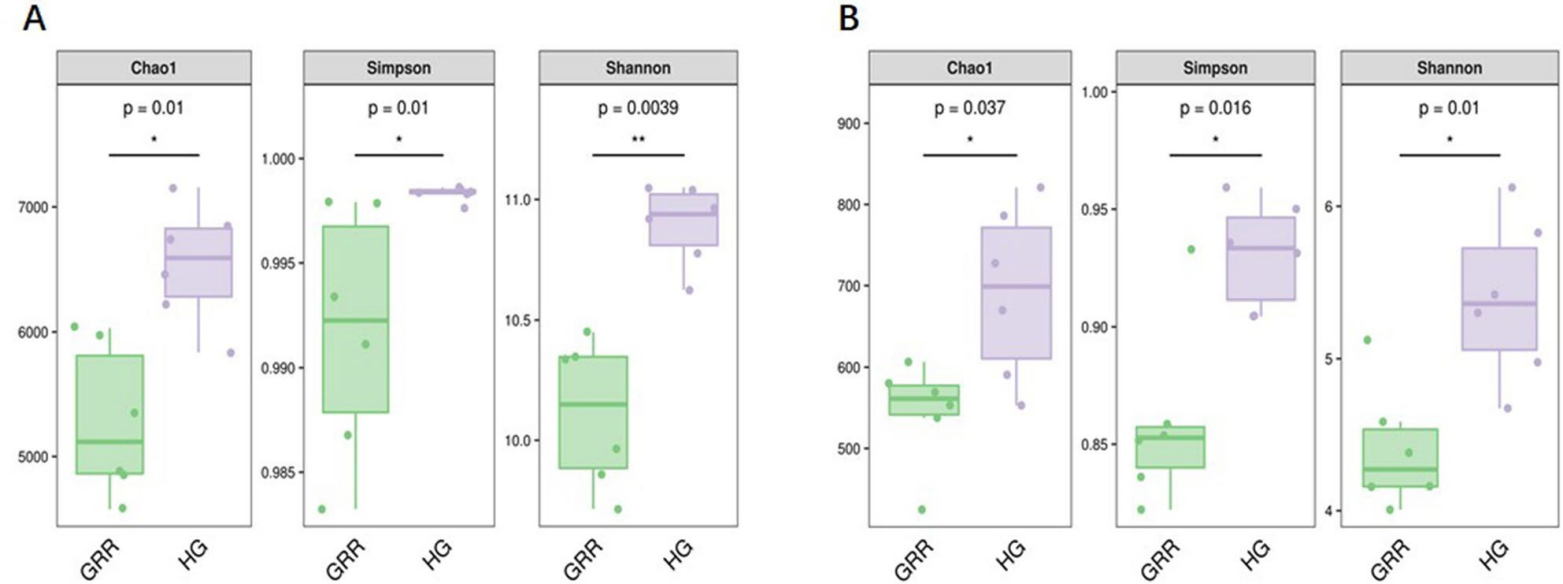
Microbial richness and diversity. (**A**) Bacteria; (**B**) fungi. HG: healthy ginseng soil group; GRR: ginseng rusty root symptom soil group. Letters indicate significant differences among different soil samples by ANOVA at *P* * < 0.05, *P* ** < 0.01 versus HG group (n = 6 in each group).

The GRR and HG’s microbial diversity was characterized by partial 16S and ITS rRNA gene sequencing obtained from DNA directly extracted from soil samples. Rarefaction curves of observed amplicon sequence variants (ASVs) for each sample were produced at the ASV level (Fig. S2). The curves of most of the samples reached close to the plateau, indicating that our sequencing depth was able to capture most bacterial species of ginseng rhizosphere soil microbiota. Compared with the rhizosphere soil of HG, the alpha diversity (including Chao1, Simpson, and Shannon diversity) of bacteria and fungi community in rhizosphere soil of GRR decreased significantly (Fig. 4). In other words, compared with the healthy ginseng rhizosphere soil, the microbial richness of GRR rhizosphere soil decreased significantly, whether it was bacteria (*P* < 0.05) or fungi (*P* < 0.05). Besides, the comparison between the two groups of samples on Simpson and Shannon index showed that the diversity of bacteria and fungi in the rhizosphere soil of GRR also decreased significantly (*P* < 0.05).

**Figure 5:**
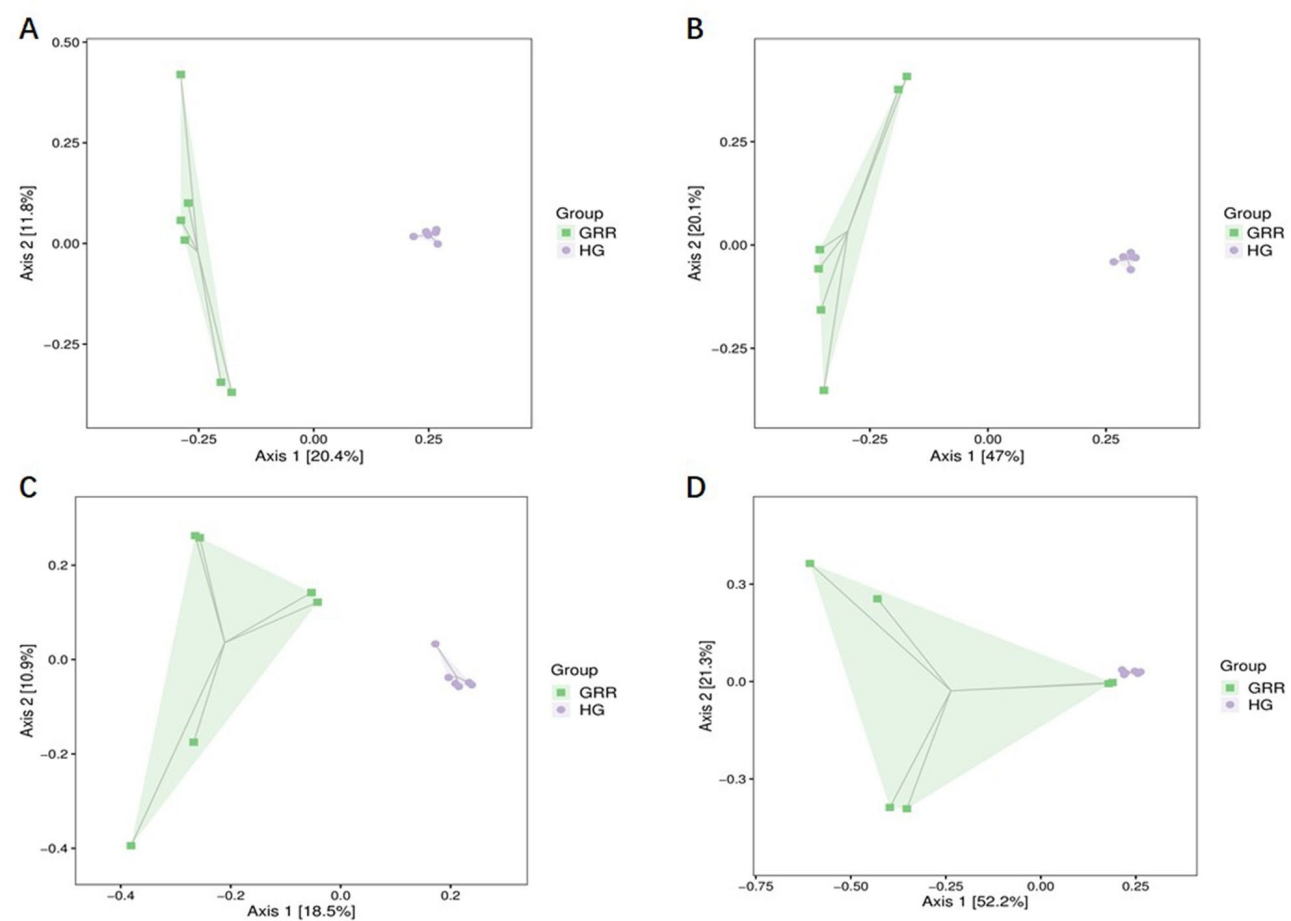
Microbial principal coordinate analysis (PCoA). (**A**) Bacteria Jaccard distance; (**B**) bacteria Bray–Curtis distance; (**C**) fungi Jaccard distance; (**D**) fungi Bray–Curtis distance. HG: healthy ginseng soil group; GRR: ginseng rusty root symptom soil group (n = 6 in each group).

According to the principal coordinate analysis (PCoA) based on Jaccard and Bray–Curtis matrix, the microbial community structure of rhizosphere soil is significantly different in HG and GRR (Fig. 5). Analysis of HG and GRR rhizosphere soil bacteria revealed a significant separation between the Jaccard (Fig. 5A) and Bray Curtis (Fig. 5B) matrices, indicating substantial differences in bacterial composition between the two groups. In the fungi analysis, except that two samples in the GRR group in Fig. 5D are close to HG, other samples also show apparent separation. The above results show that the composition of the two groups of fungi is also different.

Interestingly, based on the Jaccard or Bray–Curtis difference matrix, the samples of the HG group were highly clustered, while the samples of the GRR group were highly separated. In other words, the rhizosphere soil microbial community of HG is relatively stable, and the microorganism of GRR rhizosphere soil showed even differences.

### Species difference analysis and biomarkers of GRR and HG rhizosphere soil

**Figure 6:**
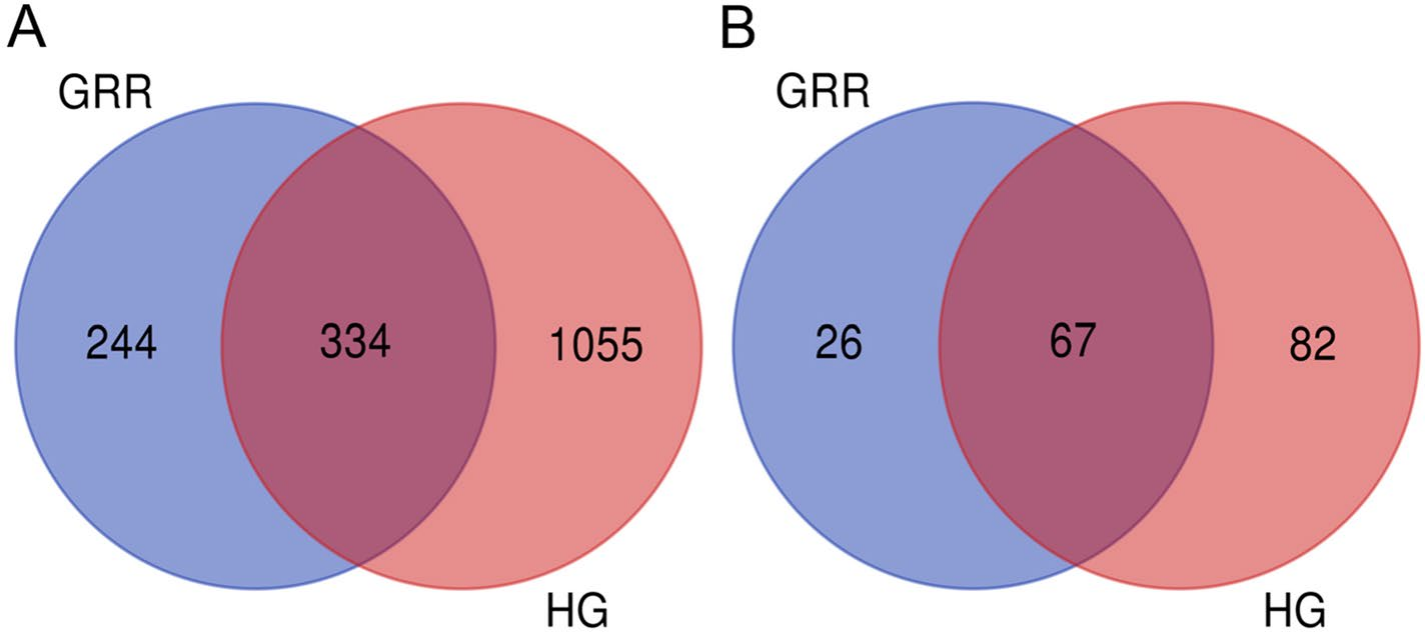
Venn diagrams of ASVs among soil samples. (**A**) Bacteria; (**B**) fungi. HG: healthy ginseng soil group; GRR: ginseng rusty root symptom soil group.

By sequencing, we obtained 33,798 ASVs in bacteria and 3,225 ASVs in fungi. We carried out the Venn map analysis of identify the potential biomarkers (taxa with stable significant differences between groups) of bacteria and fungi in two groups of soil samples. As shown in Fig. 6A, in bacteria, the number of ASVs present in all six samples in the GRR group was 578, and the number of ASVs present in all samples in the HG group was 1,389. Two groups of common ASVs are 334. Compared with HG rhizosphere soil, the total ASVs of GRR rhizosphere soil decreased, and the specific ASVs increased. In fungi, the 67 ASVs are common to all the samples, 26 specific ASVs in the GRR group, and 82 specific ASVs in the HG group (Fig. 6B). Thus, there was also a large difference in fungi ASVs between the rhizosphere soils of GRR and HG.

**Figure 7:**
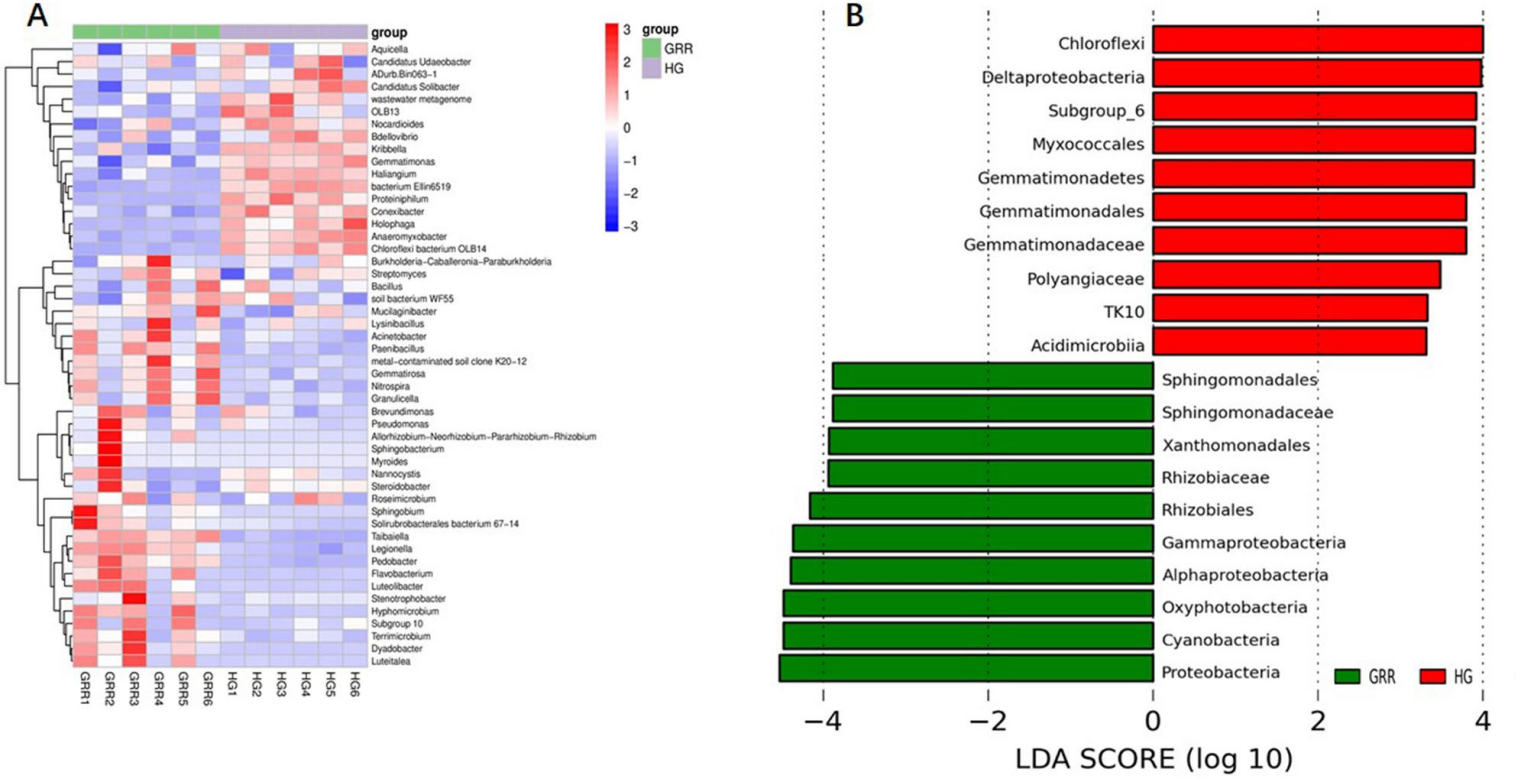
Bacterial abundance and biomarkers. (**A**) Heatmap of the 50 most abundant genera in soil samples; (**B**) histogram of LDA effect value of the histogram of LDA effect value of differentially abundant taxa. HG: healthy ginseng soil group; GRR: ginseng rusty root symptom soil group.

In this study, we used the abundance data of the top 50 genera of the average abundance of bacteria to draw a heatmap (Fig. 7A). Then, we use the histogram of LDA effect value of biomarker species based on linear discriminant analysis (LDA) effect size (Lefse) analysis to show the bacterial taxon with a significant difference between groups, and Fig. 7B shows the 20 taxa with the most significant differences between the two groups (All significantly different taxa are provided in Fig. S3). Compared with HG rhizosphere soil, the abundance of Proteobacteria, Cyanobacteria, Oxyphotobacteria, Alphaproteobacterial, Gammaproteobacterial, Rhizobiales, Xanthomonadales, and Sphingomonadales in GRR rhizosphere soil significantly increased. On the other hand, the Deltaproteobacteria; Chloroflexi; Myxococcales; Gemmatimonadetes; Gemmatimonadales; Gemmatimonadaceae; Polyangiaceae; TK10; Acidimicrobiia is more abundant in HG groups compared to GRR groups. Besides, the cladogram of intergroup difference taxa based on Lefse analysis is provided in Figure S4.

**Figure 8:**
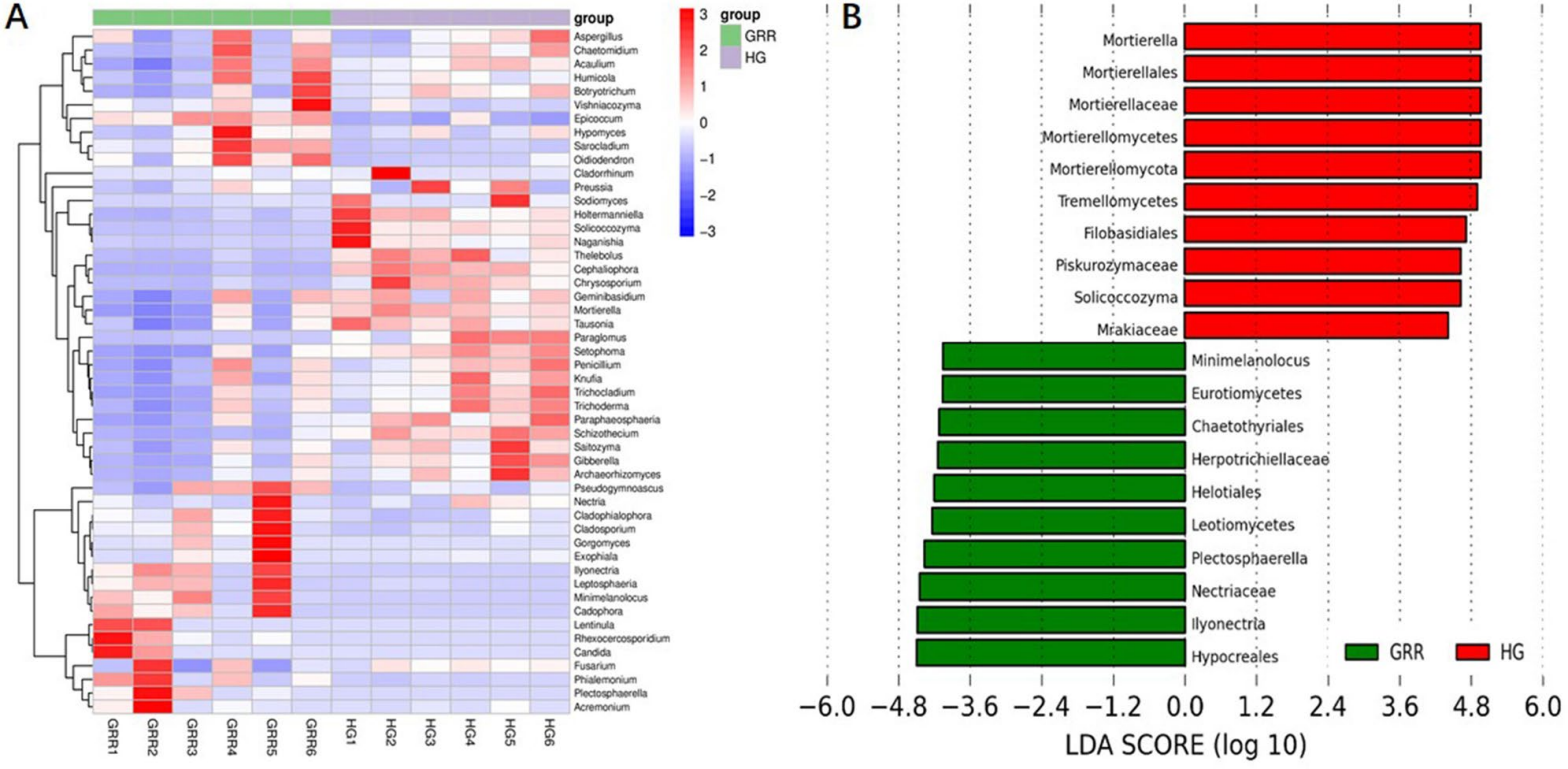
Fungi abundance and biomarkers. (**A**) Heatmap of the 50 most abundant genera in soil samples; (**B**) histogram of LDA effect value of differentially abundant taxa. HG: healthy ginseng soil group; GRR: ginseng rusty root symptom soil group.

In the fungi analysis, we also used the abundance data of the first 50 genera of average abundance to make the heatmap (Fig. 8A). The histogram of the LDA effect value of 20 biomarkers, with the most significant difference between the two groups, is shown in Fig. 8B. Among them, the 20 most significant taxa of the GRR group are *Minimelanolocus*, Eurotiomycetes, Chaetothyriales, Herpotrichiellaceae, Helotiales, Leotiomycetes, *Plectosphaerella*, Nectriaceae, *Ilyonectria* and Hypocreales. The most apparent ten taxa in the HG group are Mortierellaceae, Mortierellomycota, Mortierellomycetes, Tremellomycetes, Filobasidiales, Piskurozymaceae, *Solicoccozyma*, and Mrakiaceae (all significantly different taxa are provided in Fig. S3). The cladogram of the intergroup differences in fungi is presented in Figure S4.

### Relationship of soil physicochemical properties and enzyme activity to microbial abundance

**Figure 9:**
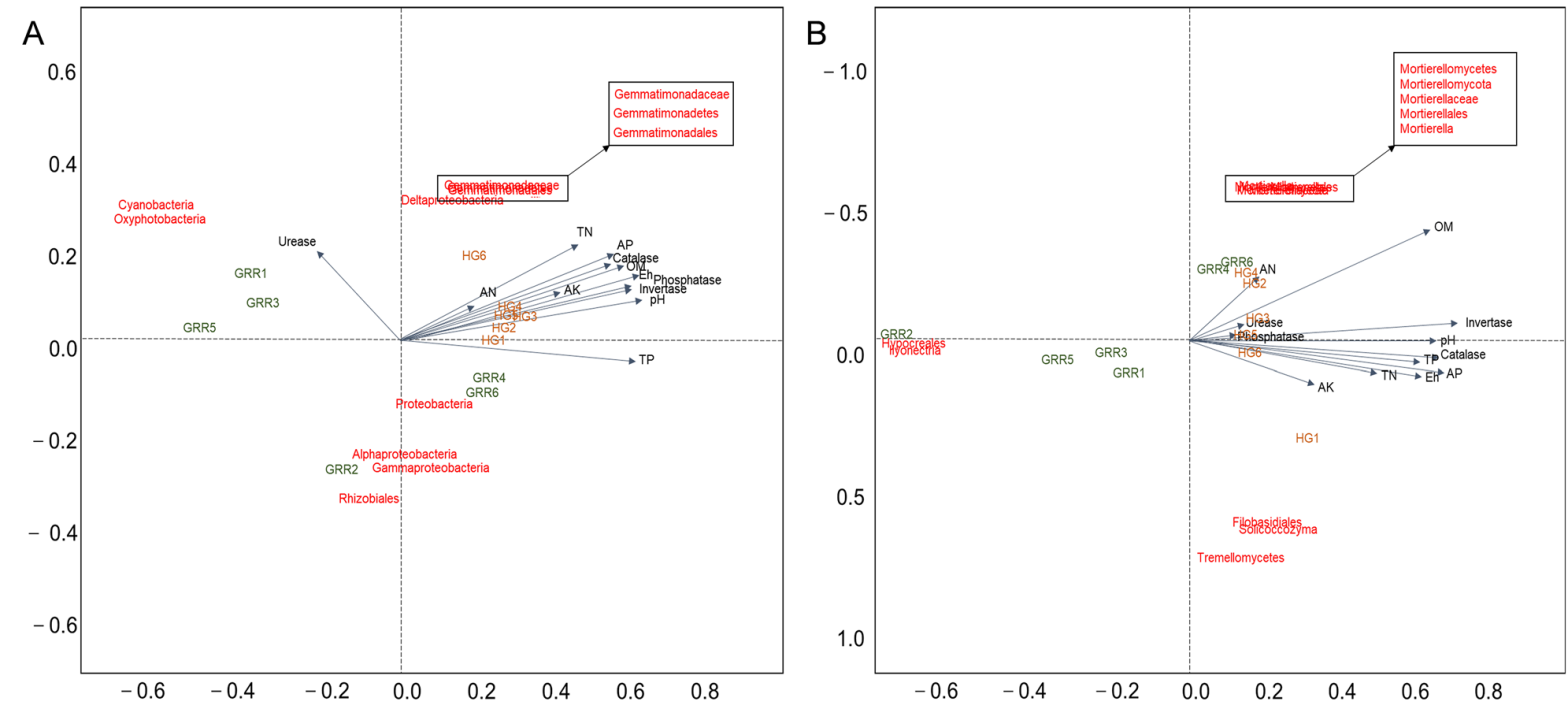
Redundancy analysis (RDA) of soil samples; (**A**) RDA plots of bacteria in soil samples; (**B**) RDA plots of fungi in soil samples. HG: healthy ginseng soil group; GRR: ginseng rusty root symptom soil group.

In redundancy analysis (RDA), we used measured soil physicochemical properties and multiple enzyme activities as environmental variables to investigate their correlation with high relative abundance taxa in soil samples. The analysis results show that bacteria and fungi in two rhizosphere soils are greatly influenced by physicochemical properties and enzyme activities. Furthermore, RDA plots reflect the relationship between several beneficial or pathogenic microorganisms and environmental variables. In the analysis results of bacteria, only Cyanobacteria and Oxyphotobacteria showed a positive correlation with Urease. Gemmatimonadaceae, Gemmatimonadetes and Gemmatimonadales are positively correlated with a variety of environmental variables. Proteobacteria, Alphaproteobacteria, Rhizobiales, Gammaproteobacteria and Deltaproteobacteria have not shown a strong correlation with various environmental variables (Fig. 9A). In fungi, *Ilyonectria* and Hypocreales were negatively correlated with various environmental variables. Mortierellaceae Mortierellomycota, Mortierellomycetes Mortierellales and Mortierella were positively related to the AN and OM. However, there is no strong correlation between Solicoccozyma, Tremellomycetes and Filobasidiales and environmental variables.

## Discussion

In this study, GRR rhizosphere soil and HG rhizosphere soil were compared comprehensively and carefully. Most of the previous studies were based on soil samples from different ginseng farms in different regions^10^. To reduce the influence of unknown differences, the soil samples collected are from the same ginseng farm with a high rusty root index. To study the relationship between ginseng rusty root and soil, this study compared the soil physical and chemical properties and nutrient content, enzyme activity, and microbial community. This study examined the soil physical and chemical properties, nutrient content, enzyme activity, and microbial community to study the relationship between ginseng rusty root and soil.

Soil pH seriously affects crop growth and microbial community structure in the soil^11,12^. Compared with healthy ginseng rhizosphere soil, the pH of GRR rhizosphere soil decreased significantly (Fig. 1A). Ginseng in northern China usually grows in acid soil, but the lower pH value may affect the healthy growth of ginseng. The pH has a particular influence on microbial community structure, diversity, and richness^13,14^. Therefore, fungi or bacteria related to GRR may appear in the soil with pH changes. There are many redox systems in soil, such as oxygen systems, iron system, manganese system. Under certain conditions, each soil has its Eh value^15^. In this study, Eh value in GRR soil decreased significantly compared with that in HG soil (Fig. 1B). Its higher water content may cause the change of the Eh value of GRR soil, or some chemical reactions may occur in the soil. For a long time, high soil moisture content has been considered one of the factors causing GRR, and the part of this rust root is deemed to be the product of some chemical reactions^2^.

The growth of ginseng has high requirements for various substances in the soil. Phosphorus is an essential component of nuclear and membrane structure in plants. Normal phosphorus levels can complete the healthy metabolism of protein in plants, stimulate the growth of plant roots, increase the absorption of mineral nutrients by rhizomes, to alleviate the damage of plant diseases^16,17^. In our results, GRR rhizosphere soil phosphorus significantly decreased compared with HG rhizosphere soil (Fig. 2C, E). Besides, potassium can affect the metabolism of plants. Furthermore, potassium can promote the development of the thick outer wall of epidermal cells, thus preventing the occurrence of diseases^16,19^. As shown in Fig. 2F, the GRR rhizosphere soil had significantly less potassium than the HG rhizosphere soil. This result seems to imply that GRR is closely related to the potassium content of the soil. Overall, the decrease of some nutrients in GRR soil may affect the healthy growth of ginseng. The GRR may be related to reducing plant disease resistance caused by the reduction of phosphorus and potassium in the soil.

Soil enzymes are the products of residual decomposition of plants and animals, exudation of plant roots, and metabolism of soil microorganisms, which involve many critical biochemical processes in soil^20^. Soil enzyme activity is an essential index of soil fertility, quality, and health^21^. The catalase, invertase, urease and phosphatase activities in rhizosphere soil of ginseng were measured in this experiment. The results showed that except urease, the activities of the other three enzymes in ginseng rhizosphere soil decreased significantly (Fig. 3). Urease catalyzes the hydrolysis of urea to carbon dioxide and ammonia, which plays an essential role in the nitrogen cycle in soil^22^. In our results, it did not show a significant correlation with GRR. Invertase is a ubiquitous enzyme in soil, which plays an essential role in the release of fructose and glucose from sucrose and provides a carbon source for the growth of soil microorganisms^23^. The organic phosphorus in the soil can be absorbed and utilized by crops under the action of phosphatase. Therefore, soil phosphatase activity can be an essential indicator of the hydrolysis of soil phosphorus compounds^24^. The decrease of phosphorus in GRR soil may be related to the reduction of the enzyme activity. Catalase can drive the decomposition and transformation of peroxides in soil, eliminating the adverse effects of peroxides on soil quality^25^. In other words, the decrease of catalase activity in GRR soil may make ginseng suffer more oxidative stress^26^. In brief, the reduction of these three enzymes in GRR soil indicated that the production of ginseng rusty root might be related to the decrease of carbon source and available phosphorus, and the content of peroxides in the soil.

In this study, high-throughput sequencing technology was used to study the microbial diversity of GRR rhizosphere soil. The alpha diversity analysis of GRR and HG rhizosphere soil, the Chao1 estimator^27^, Shannon^28^, and Simpson^29^ diversity of ginseng rhizosphere soil with rusty root showed a significant decline (Fig. 4). Therefore, in this study, the richness and diversity of fungi or bacteria in the rhizosphere soil of GRR significantly decreased. Then, we conducted the beta diversity analysis based on PCoA, Jaccard, and Bray–Curtis distance was estimated in this study (Fig. 5). Jaccard distance and Bray–Curtis distance are measures used to measure the difference in species composition of different soil samples. They can calculate the characteristics of different species composition of samples. The focus of the two analyses is different: Jaccard only considers the presence or absence of species in the sample, while Bray–Curtis considers the presence or absence of species in the sample and considers the relative richness of different species. Whether fungi or bacteria, the GRR group, and HG group had visible separation, which indicated that GRR rhizosphere soil microorganism had significant changes compared with HG soil microorganism. It should be noted that six samples of the HG group showed high aggregation. In comparison, six samples of the GRR group were very dispersed, which indicated that the microbial structure of HG rhizosphere soil was very stable. In contrast, that of GRR rhizosphere soil might have microbial community disorder. Finally, we analyzed the species that caused these differences. Using the ASVs abundance table to make Venn diagrams for community analysis shows the ASV level variation between the two groups (Fig. 6). To further compare the differences in species composition between samples, we use the abundance data of the top 50 genera of average abundance to draw a heatmap to display the genera abundance distribution trend of each sample (Figs. 7A, 8A). In this study, LEfSe analysis was used to find the biomarkers between groups, and LDA effect histograms and cladograms were drawn for bacteria and fungi, respectively (Figs. 7B, 8B and Fig. S4). We found possible pathogens in the GRR rhizosphere soil. However, the other results of LEfSe analyse were different from previous studies, which may be related to the sample collection site and the cultivation conditions.

In terms of fungi, the biomarker’s analysis found *Ilyonectria*, which is thought to be the pathogen of GRR^30^. Hypocreales is often reported to be related to pests' pathogenicity or some bacteria^31–33^, and it can survive in harsher soil conditions^34^. Nectriaceae is also listed as a biomarker. It includes numerous important plant and human pathogens, and several species used extensively in industrial and commercial applications as biodegraders and biocontrol agents^31^. We found a possible pathogen *Plectosphaerella*, it has been reported to be associated with plant root diseases^35^, but it has not yet been reported on ginseng disease. In HG rhizosphere soil, Mortierellales, Tremellomycetes orders predominated. *Mortierella* is a biomarker in HG rhizosphere soil, but rarely in GRR soil. *Mortierella* is the producer of polyunsaturated fatty acids^36^, whether it is related to ginseng growth has not been reported. There are reports that Tremellomycetes could efficiently act as opportunists that utilize the decomposition products provided by other microbes^37^. RDA reveals the correlation between fungal taxa and environmental variables (Fig. 9B). *Ilyonectria* was negatively correlated with several environmental variables, especially pH, catalase, and invertase.

On the other hand, in the analysis of bacteria, more proteobacteria appeared in GRR rhizosphere soil. Although proteobacteria have been reported to dominate pesticide and herbicide contaminated soils^38,39^, it is generally one of the most abundant phyla in many soils and rhizosphere soils. The reasons for the increase of proteobacteria in GRR rhizosphere soil need to be further explored. A significant increase in Proteobacteria often indicates the dysbiosis in the microbiota. Further, a higher abundance of Alphaproteobacterial (including Sphingomonadales) and Gammaproteobacterial (including Xanthomonadales) indicates that GRR rhizosphere soil may have stronger nitrification and the turnover of OM^40^. Interestingly, anaerobic Deltaproteobacteria (including Myxococcales) is more abundant in HG rhizosphere soil^41^. Myxococcus is considered to be an essential and active predator that regulates bacterial communities in agricultural land^42^. Cyanobacteria are the main constituent of biological bacteria and can survive in more extreme environments^43^. Some cyanobacteria have increasing soil stability and moisture-holding capacity^44,45^. Their abundance increased in GRR rhizosphere soil, confirming the deterioration of GRR rhizosphere soil conditions compared to HG. Chloroflexi (including TK10) is abundant in HG rhizosphere soil. It seems to play a useful role in the soil in providing the filamentous scaffolding around which flocs are formed feed on the debris from lysed bacterial cells. It can ferment carbohydrates and degrade other complex polymeric organic compounds to low molecular weight substrates to support their growth and that of different bacterial populations^46^. Also, Acidobacteria subgroup 6 and Gemmatimonadetes emerged as the keystone taxa in HG rhizosphere soils, and they may play an important ecological role by degrading polysaccharides of plant and fungal origin^47,48^. These differences could be partially due to the different physicochemical characteristics of the two soils^49^. Fungal plant pathogens live in a specific environment, and the GRR rhizosphere soil environment may be more suitable for the survival of pathogenic fungi such as *Ilyonectria*.

Many studies have pointed out that ginseng, American ginseng (*Panax quiquefolium* L), (*Panax notoginseng* (Burkill) F. H. Chen ex C. H.), and other medicinal plants have rusty root symptoms^30,50^. Furthermore, they all seem to be closely related to *Ilyonectria*. The same results were found in our study. However, in Wang et al.^51^, *Ilyonectria* does not have a large abundance in GRR rhizosphere soil. Also, in Liu et al. research results, *Ilyonectria* is positively correlated with N and OM^10^. In our analysis, there is a negative correlation. *Plectosphaerella*, as A potential pathogen, also showed greater abundance in our results, which had not been found in the study of Liu et al. In agreement with previous studies, we also found large abundances of Proteobacteria, Gammaproteobacterial and Rhizobiales in GRR rhizosphere soil. What is different is that we also found a large abundance of Cyanobacteria. Interestingly, our analysis of HG rhizosphere soil bacterial and fungal abundance showed significant differences from previous studies. Thus, the microbial composition of the rhizosphere soil of ginseng is likely to be closely related to the planting site, cultivation method, fertilizer and pesticide application.

In conclusion, pH and Eh in GRR soil decreased significantly, which may be related to the metabolism of the ginseng root system and soil aeration. GRR also showed significant changes in soil nutrient content, reducing TP, TK, and AK, making ginseng absorb fewer nutrients and may affect the disease resistance of ginseng. The decrease in the activities of many enzymes in GRR soil indicates that the metabolism of soil substances is greatly affected. Besides, compared with HG rhizosphere soil, microbial abundance and diversity of GRR decreased significantly, community structure also changed dramatically, and community disorder occurred. Finally, through Lefse analysis, the number of microorganisms in GRR rhizosphere soil that may be beneficial to the soil decreased, and the pathogenic bacteria (*Ilyonectria*) mentioned in previous studies were also found. Soil is a complex environment. Physical and chemical properties, nutrients, and microbial communities affect the growth of ginseng. RDA analysis of the correlation between different microorganisms and environmental factors will help improve ginseng planting soil in the future. This study mainly studies the change of ginseng rusty root symptom soil, and ginseng rusty root symptom may be caused by many factors that need further investigation.

## Materials and methods

### Soil information and sample collection

All soil samples were collected from the same ginseng farm with ginseng (5-year-old) in Hunchun city, Jilin province, China (42.86′ N and 130.37′ E). In detail, this is a ginseng farm with an area of about 21,000 square meters, with an altitude of 40 m, belongs to the temperate marine climate, with an annual average temperature of 5.65 °C, average precipitation of 617.9 mm, a frost-free period of 140–160 days, and an average temperature of 21.2 °C in August. The farm was the first time to cultivate ginseng and was cultivating transplanted 2-year-old ginseng seedlings. The pesticides applied in ginseng cultivation are “carbendazim (systemic broad-spectrum fungicide)” bought from Shandong Xinnongji Biotechnology Co. LTD (China), “tiandashenbao (plant cell membrane stationary agent)” bought from Shandong Tianda Bio-pharmaceutical Co. LTD (China) and “metalaxyl (Phenylamide fungicides)” bought from Alta Technologies LTD (China). All pesticides are used strictly following the “Ginseng safe production technical specification of pesticide application (DB22/T 1233-2019)”.

We randomly collected 200 ginseng and calculated the rusty root index^9,52^ to evaluate the severity of the disease.

$$ {\text{ Rusty}}\;{\text{ root}} \;{\text{index}} = \frac{{\sum \left( {{\text{n}} \times {\text{rusty}}\;{\text{root}}\;{\text{grade}}} \right)}}{{N \times {\text{the}} \;{\text{highest}} \;{\text{rusty}} \;{\text{root}} \;{\text{grade}}}} $$

In this formula: n = The quantity of ginseng in different rusty root grades, N = Total number of ginsengs used for statistics. Rusty root grade was divided into 0–4, which were healthy ginseng root, rust area < 10%, 10–25%, 25–50%, and > 50% in turn.

Then, six plots with ginseng grade 0 and six plots with ginseng grade 4 were selected to collect soil samples, about 1 square meter per plot. The soils were sampled from each plot at depths of 5–15 cm, representing soils from the root zone and below the root zone. The 12 soil samples are divided into the HG group (healthy ginseng group) and GRR group (ginseng rusty root symptom group). Part of each soil sample is kept wet for the determination of soil Eh.

Finally, six HG rhizosphere soils and six GRR (grade 4) rhizosphere soils were collected from the 12 plots above (one rhizosphere soil sample was collected at each polt). Part of the soil microbial DNA was extracted, and the samples were kept in the sterile centrifuge tube at—80 °C until use. The other part of the soil was air-dried at ambient temperature, crushed, and sieved through a 2 mm plastic mesh to determine soil pH, soil nutrition, and soil enzyme activity^53^. In this part, only the soil attached to the ginseng root is considered as rhizosphere soil^54^.

### Analysis of basic physical and chemical properties

The pH value of soil was measured with a pH meter/potentiometer under the soil: water ratio of 1:2.5. The Eh was determined by potentiometry (China HJ 746-2015)^55^. The soil OM content was measured by the potassium dichromate external heating method. The TN was determined by Kjeldahl method^55^, and the TP was determined by alkali fusion molybdenum antimony colorimetry (China HJ 632-2011). The AP was determined by NaHCO_3_ extraction molybdenum-antimony colorimetry; the AN was determined by the alkali diffusion method; AK was determined with the fame photometric method^57^.

### Analysis of soil enzyme activity

The activities of catalase, invertase, urease, and phosphatase in the soil of ginseng were measured. The activity of catalase was determined by KMnO_4_ titration; soil invertase activity was determined by 3,5-dinitrosalicylic acid colorimetry; urease activity was determined by indophenol blue colorimetry; acid phosphatase activity was determined by disodium phenyl phosphate colorimetry method^38^.

### DNA extraction and Illumina sequencing

DNA was extracted from 0.5 g of soil (wet weight) using the Mag-Bind soil DNA kit (Omega Bio-tek, Norcross, GA, U.S.) according to the manufacturer’s protocols. Then, DNA was quantified by Nanodrop (Thermo Scientific, Waltham, MA, U.S.) and the quality of DNA was detected by 1.2% agarose gel electrophoresis, the OD_260/280_ values of DNA extraction were between 1.8 and 2.0.

The V3-V4 region of bacterial 16s rRNA gene was amplified by bacterial specific primers 338F and 806R, and the internal transcribed spacer (ITS1) was amplified by eukaryotic specific primers ITS5F and ITS1R. In this step, the sample is uniformly diluted to 20 ng/ul. Amplification system (25 μL): 5 × reaction buffer 5 μL, 5 × GC buffer 5 μL, Dntp (2.5 Mm) 2 μL, Forwardprimer (10 uM) 1 μL, Reverseprimer (10 uM) 1 μL, DNA Template 2 μL, ddH_2_O 8.75 μL, Q5 DNA Polymerase 0.25 μL (NEB). Amplification parameters: Initial denaturation 98 °C 2 min, Denaturation 98 °C 15 s, Annealing 55 °C 30 s, Extension 72 °C 30 s, Final extension 72 °C 5 min, 10 °C Hold. 25–30 Cycles. The sequencing library was prepared by using Illumina’s TruSeq Nano DNA LT Library Prep Kit, the final fragment was selected and purified by 2% agarose gel electrophoresis.

The library was quantified by Quant-iT PicoGreen dsDNA Assay Kit in Promega QuantiFluor fluorescence quantitative system, and then libraries were sequenced on the Illumina HiseqPE250 platform at Personal Biotechnology Co., Ltd. (Shanghai, China).

### Bioinformatics and data analysis

Microbiome bioinformatics was performed with QIIME 2 2019.4^58^ with slight modification according to the official tutorials (https://docs.qiime2.org/2019.4/tutorials/). Briefly, raw sequence data were demultiplexed using the demux plugin following by primers cutting with cutadapt plugin^59^. Sequences were then quality filtered, denoised, merged, and chimera removed using the DADA2 plugin^60^. Non-singleton ASVs were aligned with mafft^61^ and used to construct a phylogeny with fasttree2^62^. To analyze different samples using the same sequencing depth, all samples were re-sampled into the same sequence number: 119938 sequences from fungi and 91,828 sequences from bacteria. Alpha diversity metrics: The Chao1 estimator, Simpson and Shannon diversity index. Beta diversity metrics: Jaccard distance and Bray–Curtis dissimilarity. Taxonomy was assigned to ASVs using the classify-sklearn naïve Bayes taxonomy classifier in feature-classifier plugin^63^. LEfSe was used to elucidate the biomarkers in each group^64^. In addition, RDA was used to reveal the relationships between microbiota and soil properties. This analysis was carried out with the “vegan” package in R.

### Statistical analyses

All data were analyzed using the SPSS software (IBM Corporation, Armonk, NY, USA), and the results were expressed as the arithmetic mean value ± standard deviation. The differences in the means were compared by the Student’s *t* test at *P* < 0.05.

## Supplementary information

Supplementary Legends.

Supplementary file2

Supplementary file3

Supplementary file4

Supplementary file5

Supplementary Table.
