# Supplementary material for: Comparative analysis of rhizosphere soil physiochemical characteristics and microbial communities between rusty and healthy ginseng root
Source: Sci Rep. 2020 Sep 25;10:15756. doi: 10.1038/s41598-020-71024-8 (PMC7519692; doi:10.1038/s41598-020-71024-8)
Supplement: Supplementary file 1 — Supplementary Legends. [file 41598_2020_71024_MOESM1_ESM.docx]

**Comparative analysis of rhizosphere soil physiochemical characteristics and microbial communities between rusty and healthy ginseng root**

Xingbo Bian^a^, Shengyuan Xiao^ab^, Yan Zhao^a^, Yonghua Xu^a^, He Yang^a^ and Lianxue Zhang^a*^

1. *College of Chinese Medicinal Materials, Jilin Agricultural University, Changchun, China*
2. *National& Local Joint Engineering Research Center for Ginseng Breeding and Development, Changchun 130118, China*

* Corresponding author at: College of Chinese Medicinal Materials, Jilin Agricultural University, Changchun 130118, Jilin Province, China. Tel/Fax: +86 431 84533358, E-Mail address: zlx863@163.com (LX. Zhang).

| Rusty Root Grade | 0 | 1 | 2 | 3 | 4 |
| --- | --- | --- | --- | --- | --- |
| Quantity | 18 | 6 | 24 | 44 | 108 |

**Table S1.** Statistics of rusty root grade and quantity.

**Figure S1.** Ginseng rusty root symptom.


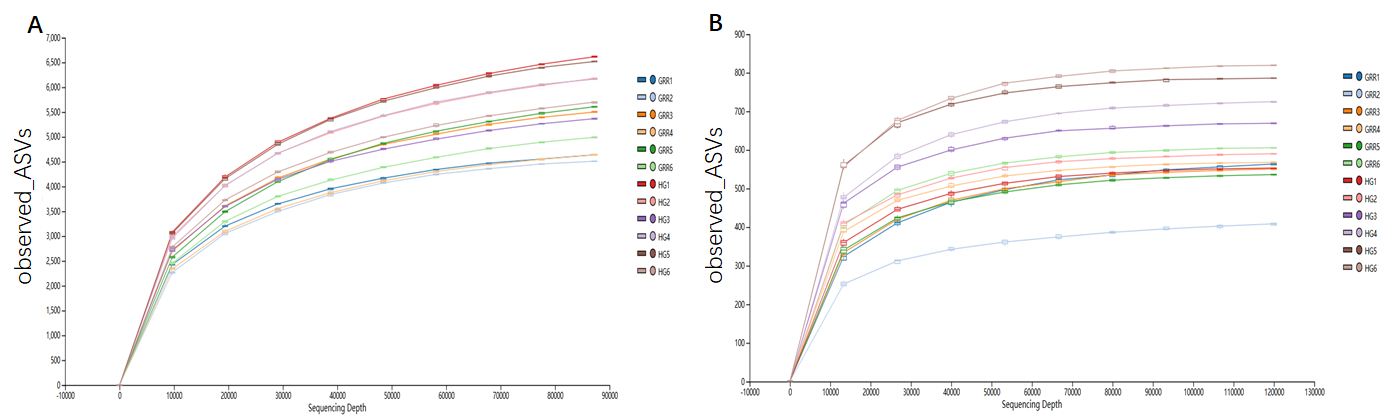


**Figure S2.** The rarefaction curves of observed ASVs at ASV level across all samples. A) bacteria; B) fungi.

**Figure S3.** The histogram of LDA effect value of differentially abundant taxa. A) bacteria; B) fungi.

**Figure S4.** Cladogram of intergroup differential taxa. A) bacteria; B) fungi.
