# Supplementary figures and images for: Comparative analysis of rhizosphere soil physiochemical characteristics and microbial communities between rusty and healthy ginseng root
Source: Sci Rep. 2020 Sep 25;10:15756. doi: 10.1038/s41598-020-71024-8 (PMC7519692; doi:10.1038/s41598-020-71024-8)

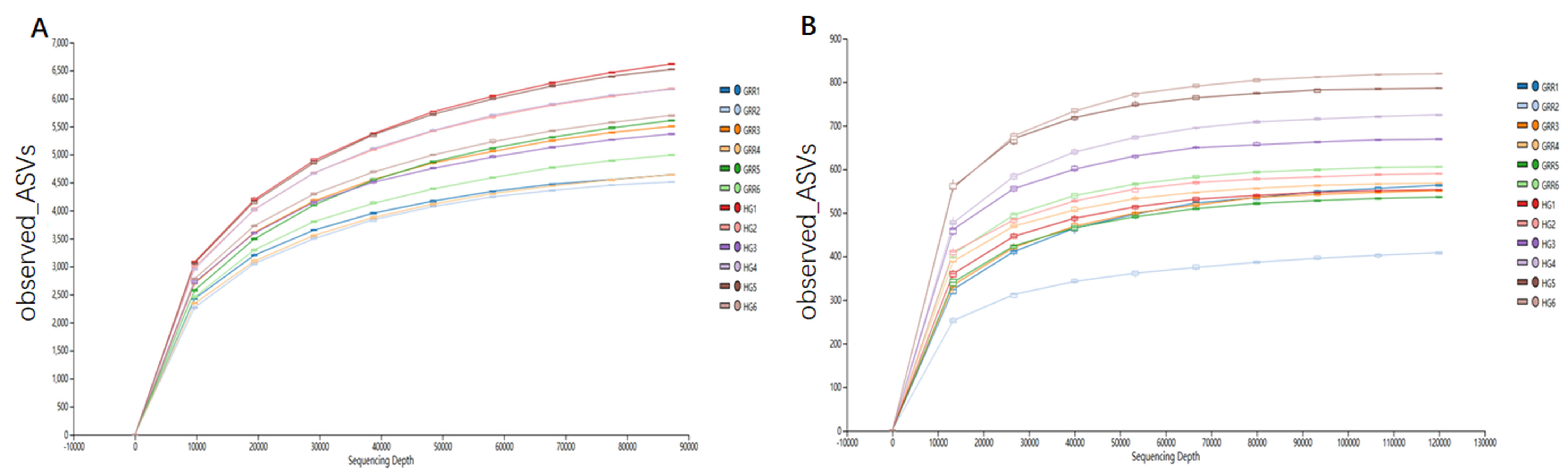

Supplement: Supplementary file 3 — Supplementary file3 [file 41598_2020_71024_MOESM3_ESM.jpg]

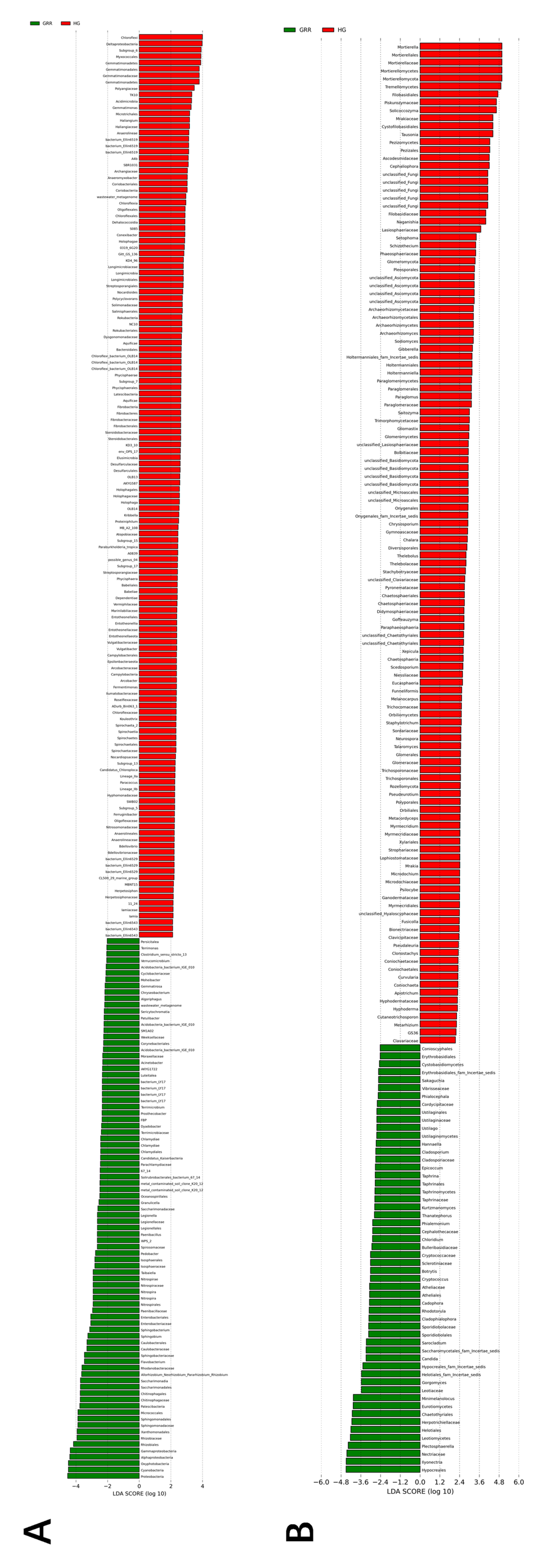

Supplement: Supplementary file 4 — Supplementary file4 [file 41598_2020_71024_MOESM4_ESM.jpg]
